# Supplementary material for: Light-regulated growth from dynamic swollen substrates for making rough surfaces
Source: Nat Commun. 2020 Feb 19;11:963. doi: 10.1038/s41467-020-14807-x (PMC7031321; doi:10.1038/s41467-020-14807-x)
Supplement: Supplementary file 1 — Supplementary information [file 41467_2020_14807_MOESM1_ESM.pdf]

Supplementary Information

Light-regulated growth from dynamic swollen substrates for making rough surfaces

Xue *et al.*

Supplementary Note1: synthesis

a) *o*-Nitrobenzyl acrylate (NBA, the promoter)

It was synthesized according to a reported procedure.[1] To a mixture of 2-nitrobenzyl bromide (5.4 g, 25 mmol) and potassium carbonate (6.9 g, 50 mmol) in DMF (60 mL), acrylic acid (8.575 mL, 125 mmol) was added dropwise. The mixture was stirred at room temperature overnight. 40 mL water was added to dissolve the insoluble salts, and the mixture was extracted with 100 mL ethyl acetate. The organic layer was washed with brine and dried over anhydrous Na_2SO_4. After column chromatographic separation (silica gel, dichloromethane), a yellow liquid product was obtained (yield: 82%).

^1H NMR (200 MHz, $CDCl_3$) δ: 7.96-8.03 (d, 1H), 7.50-7.60 (t, 2H), 7.35-7.45 (d, 1H), 6.35-6.48 (d, 1H), 6.10-6.25 (t, 1H), 5.80-5.90 (d, 1H), 5.55 (s, 2H) ppm. ^{13}C NMR (200 MHz, $CDCl_3$) δ: 165.4, 147.5, 133.7, 132.1, 131.9, 128.9, 127.7, 125.0, 62.9 ppm. LC-MS (m·z^-1): Calcd for $[M+NH_4]^+$: 255.0, Found: 255.0.

b) Linear poly(HBA-*co*-NBA)

Poly(HBA-*co*-NBA) was synthesized by reversible addition-fragmentation chain-transfer (RAFT) polymerization. Briefly, the mixture of 397.6 mg of HBA, 134.6 mg of NBA, 0.964 mg CPADB, and 2.83 mg AIBN in 2 mL of anhydrous dimethylformamide (DMF) was purged with Argon for 30 min. The initial molar ratio of HBA, NBA, CPADB, and AIBN was 160: 40: 1: 0.2. Then the mixture was sealed and heated up to 70 °C for 24 h for polymerization. The polymers were obtained by precipitating the solutions in cold diethyl ether, then dissolved in 1 mL DMF and precipitating in cold diethyl ether (three cycles), followed by drying in a vacuum.

c) 4-Hydroxybutyl acetate (HB acetate)

It was synthesized *via* a protocol previously reported.[2] An oven-dried round bottom flask was charged with a magnetic stir bar. To this flask, 1,4-butanediol (8.8 mL, 98 mmol), acetate acid (5.6 mL, 98 mmol) and DCM (60 mL) were added. Then 2 drops of H_2SO_4 was added into the above solution. The mixture was stirred for 18 h and then water was added. After that, the mixture was extracted with DCM and the organic layer was washed with a saturated solution of Na_2CO_3 and brine, dried over Na_2SO_4. After

column chromatographic separation (silica gel, dichloromethane: ethyl acetate=10: 1, *v*: *v*), the final product was given as a colorless oil (yield: 56%).

^{1}H NMR (200 MHz, $CDCl_3$) δ: 4.10-4.20 (t, 2H), 3.63-3.72 (t, 2H), 2.05 (s, 3H), 1.50-1.74 (m, 4H) ppm. ^{13}C NMR (200 MHz, $CDCl_3$) δ: 171.3, 64.3, 62.1, 29.0, 25.0, 20.9 ppm. LC-MS (m·z^-1): Calcd for $[M+NH_4]^+$: 150.0, Found: 150.1.

d) Bis-*N*, *N*'-6-hydroxyhexanol perylenetetracarboxylic diimide-acrylate (PDIDA)

Bis-*N*, *N*'-6-hydroxyhexanol perylenetetracarboxylic diimide-acrylate (PDIDA) was synthesized *via* a previously reported method.[3] Briefly, 3,4,9,10-perylenetetracarboxylic dianhydride (0.5 g, 1.295 mmol), 6-aminohexanol (0.47 g, 4.014 mmol) and imidazole (3 g) were placed in a round bottom flask and heated at 130 ^{o}C for 4 h under N_2 atmosphere. The reaction mixture was diluted with ethanol and the resulting dark red solution was filtered to remove the undissolved substance. The filtrate was acidified with 2 M HCl aqueous solution to generate red precipitate. The precipitate (bis-*N*, *N*'-6-hydroxyhexanol perylenetetracarboxylic diimide, OHPDI) was collected by vacuum filtration, washed with water until the filtrate was neutral and dried at 75 ºC overnight in a vacuum oven (yield: 61%).

^{1}H NMR (200 MHz, $CDCl_3$) δ: 8.35-8.65 (d, 8H), 3.75-4.46 (m, 8H), 1.50-1.90 (m, 16H) ppm. ^{13}C NMR (200 MHz, $CDCl_3$) δ: 165.2, 135.5, 132.9, 129.1, 126.1, 124.1, 68.8, 40.4, 30.8, 27.7, 26.2, 25.0 ppm. LC-MS (m·z^-1): Calcd for $[M+NH_4]^+$: 608.1, Found: 608.2.

OHPDI (60.8 mg, 0.1 mmol), triethylamine (72 µL, 0.5 mmol) and dry $CHCl_3$ (20 mL) were added in a round bottom flask under N_2 atmosphere. Acrylyl chloride (52 µL, 0. 5 mmol) was dissolved in 10 mL $CHCl_3$ and added dropwise into the above solution. After stirred at room temperature for 24 h, the mixture was washed with water and brine. After column chromatographic separation (silica gel, dichloromethane: methanol=20: 1, *v*:*v*), the product (PDIDA) was obtained as a red solid (yield: 30%).

^{1}H NMR (200 MHz, $CDCl_3$) δ: 8.35-8.68 (d, 8H), 6.36-6.48 (d, 1H), 6.07-6.15 (t, 1H), 5.71-5.85 (d, 1H), 3.83-4.40 (m, 8H), 1.49-1.90 (m, 16H) ppm. ^{13}C NMR (200 MHz, $CDCl_3$) 165.3, 159.1, 134.1, 131.3, 130.2, 129.5, 127.5, 125.8, 123.9, 63.5, 44.7, 31.2, 30.9, 28.6, 25.3 ppm. LC-MS (m·z^-1): Calcd for $[M+NH_4]$+: 716.2, Found: 716.1.

Supplementary Note 2: main molecules used for growth

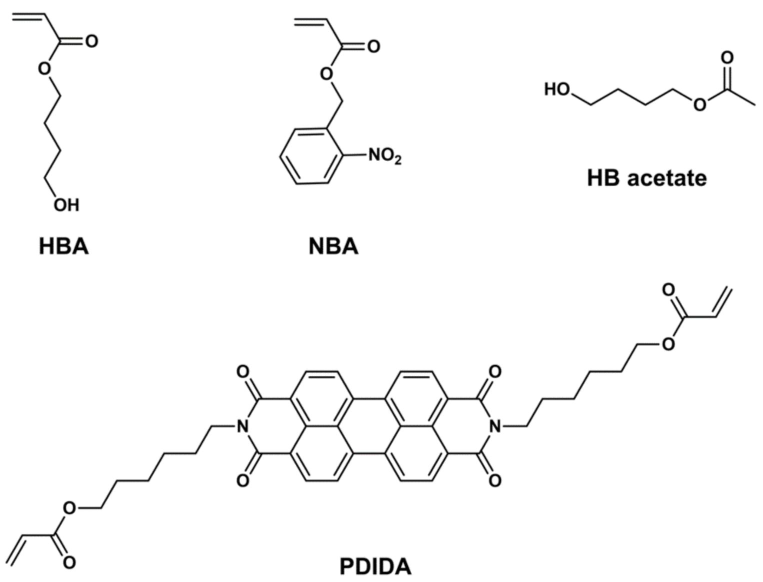

Supplementary Figure 1. Main molecules used in our concept.

Supplementary Note 3: photostability of NBA under blue light

As *o*-nitrobenzyl-ester-based monomers could be photocleaved when exposed to UV light, we should find a suitable light source for substrate curing without inducing photolysis of the NBA. Since *o*-nitrobenzyl-ester-based products have high adsorption in the wavelength (λ) of 250-350 nm, we chose the blue light (460 nm) with an intensity of 0.6 mW·cm^-2 as the light source to trigger the polymerization. UV-Vis and ^{1}H NMR spectroscopies had been utilized to detect the stability of NBA exposed to blue light (Supplementary Figure 2). The enclosed as-prepared NBA solutions (in acetonitrile and DMSO-$_{d6}$ for UV-Vis spectrum and NMR spectra, respectively) were put directly under blue light irradiation to detect its photostability in this wavelength.

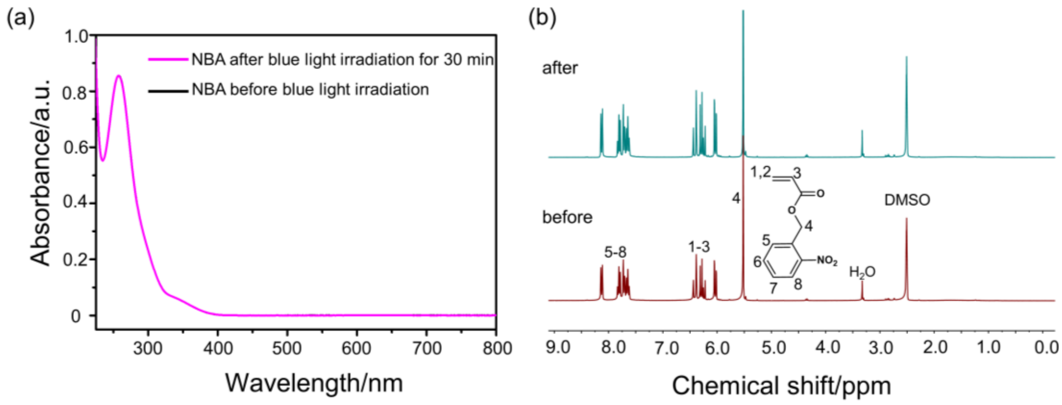

Supplementary Figure 2. UV-Vis (a) and ^{1}H NMR spectra (b) of NBA before and after blue light irradiation. The black and red lines are overlapped in (a). For the UV-Vis spectra, the concentration of the NBA in acetonitrile is 1.27×10^{-4} M. DMSO-$_{d6}$ was used for the NMR test.

Supplementary Note 4: characterization of poly(HBA-*co*-NBA)-based seeds

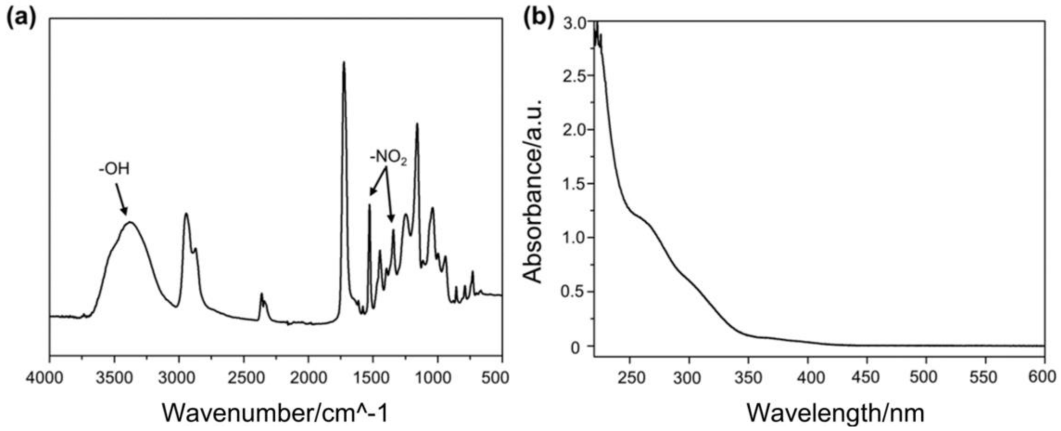

Supplementary Figure 3. ATR-FTIR (a) and UV-Vis spectra (b) of poly(HBA-*co*-NBA) based substrates.

Supplementary Note 5: swelling of seeds before and after UV irradiation

The seed-20% was immersed in the nutrient solutions consisting of HBA (96 wt%), HDDA (1 wt%), I-819 (1 wt%), and BZSA (2 wt%) for swelling. The weights of the samples with a thickness of 1.4 mm were collected to plot the swelling kinetics. Here the swelling ratio is calculated in supplementary equation (1):

$$Swelling\ ratio = \frac{W_{swollen} - W_{dry}}{W_{dry}} \quad (1)$$

$W_{swollen}$ is the weight of the swollen sample and W_{dry} is the weight of the dry sample.

Supplementary Figure 4 shows the swelling curves of the seed-20% film before and after UV irradiation.

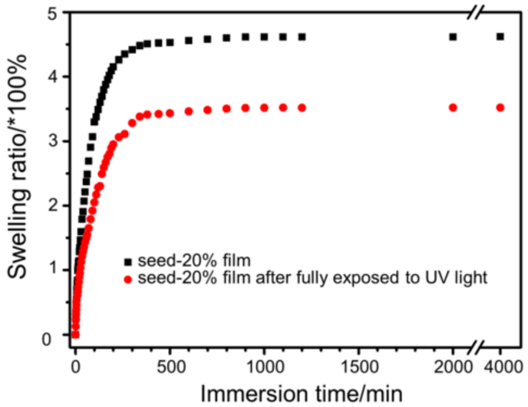

Suppleentary Figure 4. Swelling curves of a seed-20% film before and after UV irradiation in nutrient

solutions containing HBA, HDDA, I-819, and BZSA. The film was fully exposed to the UV light (10 mW·cm^-2) for 30 min before measurement.

Supplementary Note 6: photolysis of NBA units under UV light

Water contact angle was used to monitor the formation of hydrophilic groups in the substrates. It changes from 95° to 63° after UV irradiation to generate a hydrophilic group (Supplementary Figure 5a and 5b). In FTIR spectra, the peaks assigned to the group of -NO_2 (1528 cm^-1 and 1343 cm^-1) disappeared after UV irradiation (Supplementary Figure 5c).

The photolysis of NBA units to generate carboxyl side groups decreases the swelling ratio of the seeds to the nutrient solutions. Supplementary Figure 4 shows the swelling curves of the photolytic film (1.4 mm) and the equilibrium swelling ratio decreases from 4.6 to 3.5.

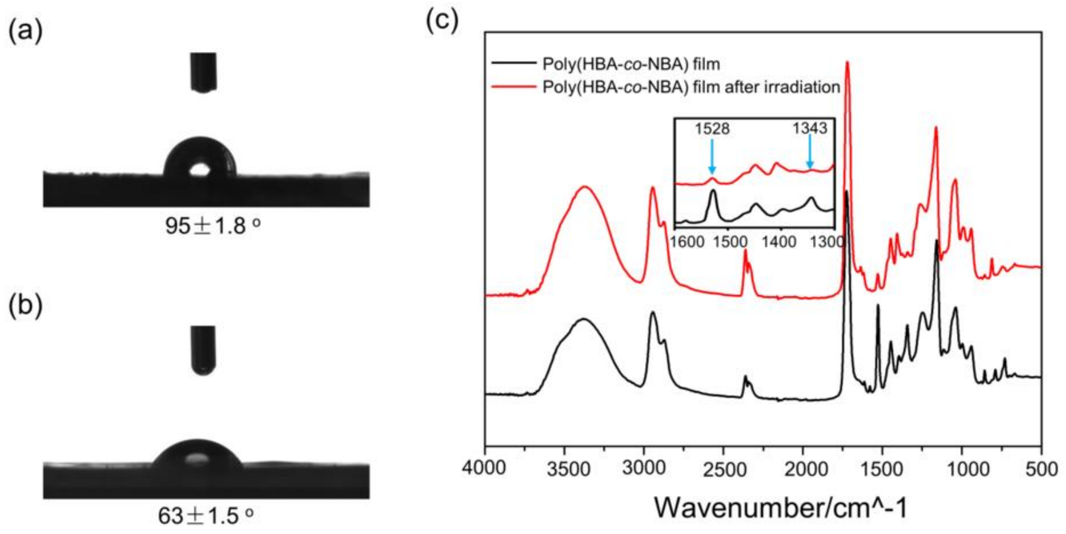

Supplementary Figure 5. Water contact angle (a and b) and FTIR spectra (c) of poly(HBA-*co*-NBA) film before and after UV irradiation for 30 min.

Supplementary Note 7: evaluation of possible UV triggered chain scission

Four kinds of control samples were subjected to UV irradiation to study the possibility of UV-triggered partial chain scission: seed-20%, swollen seed-20% containing non-polymerizable liquids, seed-0%, and swollen seed-0% containing non-polymerizable liquids. As shown in Supplementary Figure 6, after 30 min UV irradiation, the compression moduli of samples with NBA units (seed-20% sample and swollen seed-20% containing non-polymerizable liquids) slightly increase while those of non-photorespinsive samples (seed-0% sample and swollen seed-0% containing non-polymerizable liquids) do not change. These results indicated that possible UV-triggered partial chain scission was rare and its contribution to the mechanical properties was negligible.

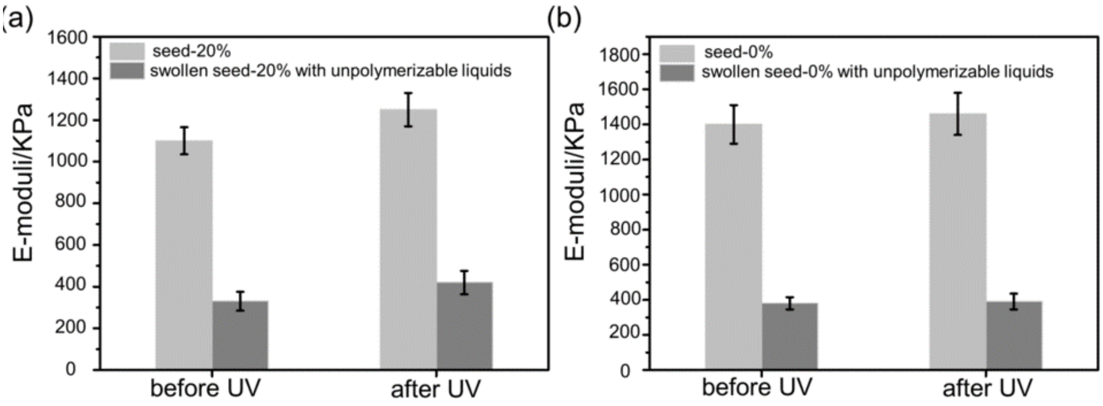

Supplementry Figure 6. Compression E-moduli of different samples before and after UV irradiation for 30 min. (a) Samples with NBA units. (b) Samples without NBA units. A 365 nm LED lamp (10 mW·cm^-2) was used for irradiation. The non-polymerizable liquid contains 4-hydroxylbutyl acetate, I-819, and BZSA.

Supplementary Note 8: polymerization of HBA/NBA/HDDA/I-819 under UV or blue light

The precursor solution used for preparing seed-20% was mixed and purged with Argon for 20 min. 50 µL of the mixture (weighted as M_0) was taken for each polymerization with different exposure times (10 s, 30 s, 1 min, 2 min, 5 min, 10 min, 20 min, 30 min, 40 min, 50 min) under 460 nm or 365 nm light irradiation (intensity: 10 mW·cm^-2). After polymerization, the unreacted components were removed by ethanol rinsing (3 times), followed by drying to obtain the crosslinked polymers (weighted as M_t). The polymerization conversion was defined as M_t/M_o. The polymerization reaches its plateau in 2 min under UV or blue light irradiation.

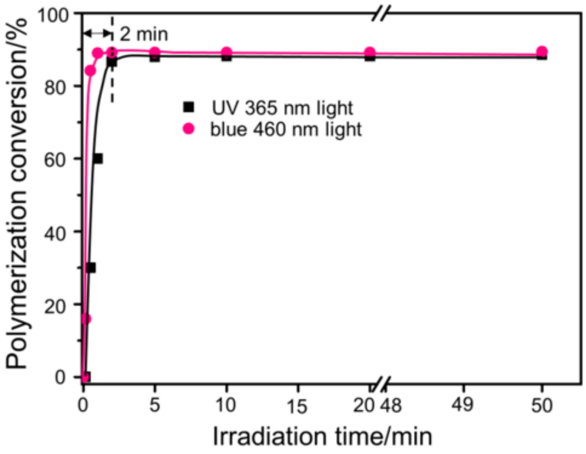

Supplementary Figure 7. Polymerization conversion of HBA/NBA/HDDA/I-819 under different irradiation conditions.

Supplementary Note 9: diffusion-induced distortion of photolysis-absent samples

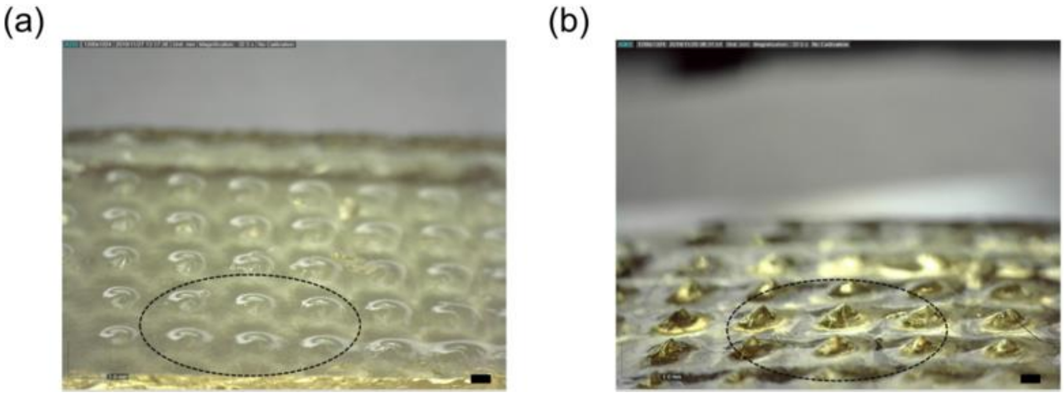

Supplementary Figure 8. Images of grown structures of photolysis-absent sample in fresh (a) and distorted (b) state. The sample in (b) was obtained *via* being stored in the dark for 32h after irradiation. Scale bar: 500 μm.

Supplementary Note 10: swelling of thin seed-20% film

A thin film with a thickness of 500 μm of poly(HBA-co-NBA) was prepared according to the protocol described in the main text. The thickness was selected because the diameter of the grown structure used for demonstration in the main text was also 500 μm. The swelling capability of this obtained seed network immersing in a nutrient solution containing HBA, HDDA, I-819 and BZSA was recorded with different treatment times. The time for the sample to reach complete swelling is nearly 4 hours without irradiation and 2 hours under irradiation. Note that here we assumed that diffusion time for liquid molecules to diffuse into the growth structure is similar to the time for a sample with a thickness of 500 μm to be completely swollen.

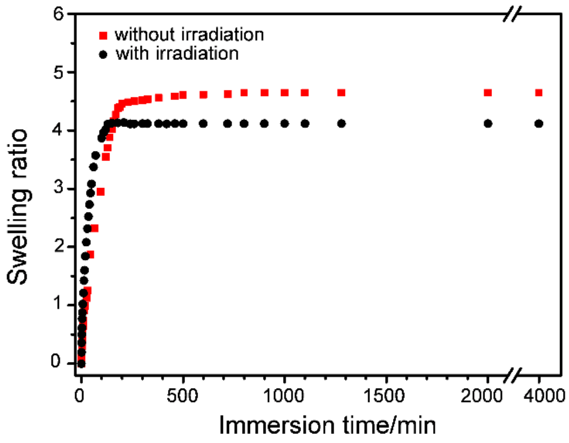

Supplementary Figure 9. The swelling ratio of seed-20% thin film (thickness: 500 μm) in the nutrient solution containing HBA, HDDA, I-819, and BZSA.

Supplementary Note 11: growth of photolysis-absent sample after being stored in dark and washing

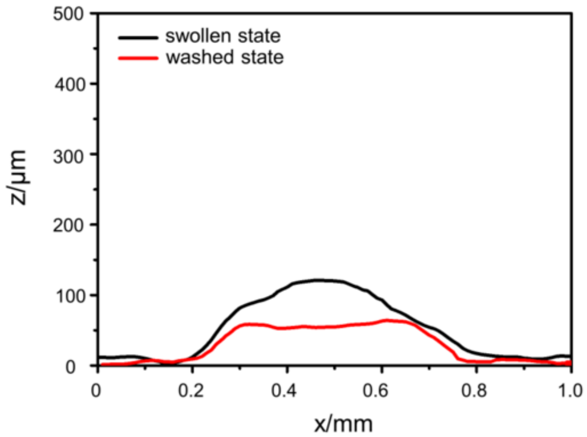

Supplementary Figure 10. Profile of grown structures of the photolysis-absent sample under different conditions. Swollen sate: sample after being stored in dark for 32 h, washed state: sample after washing by ethanol.

Supplementary Note 12: stability of seed-20% sample after growth

We evaluated the stability of the new grown structure in both swollen and washed states (Supplementary Figure 11). After being stored for 32 h, the grown structure of the sample became slightly bigger because liquid diffuses into the grown structure as a result of the concentration gradient, which could retain this shape for 1 month in dark. In the washed sample, the grown structure showed nearly the same profile even after being stored in dark for over 1 month.

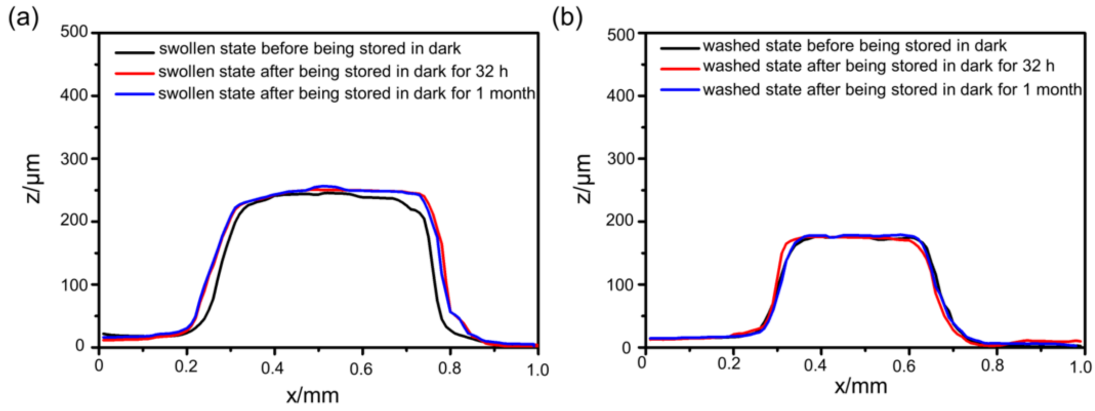

Supplementary Figure 11. Profile of grown structure from swollen seed-20% under different conditions: (a) without washing treatment, (b) with washing treatment.

Supplementary Note 13: characterization and photolysis of poly(HBA-*co*-NBA)

As shown in Supplementary Figure 12a, the copolymers contain HBA and promoters both, and the molar ratio of HBA was 22% calculated from the integration of peak c', f' and d-g , which was very accordance with the raw ratio before the polymerization. The molecular weight of the copolymers was 8500 with a PDI of 1.16 (GPC, Supplementary Figure 12b).

Supplementary Figure 13 shows the UV spectra of the poly(HBA-*co*-NBA) under UV 365 nm light irradiation. The adsorption of 265 nm decreases while that of 320 nm increases with increasing irradiation time, which indicated a sufficient cleavage of the *o*-nitrobenzyl group. The cleaving efficiency attained 100% with an energy dose of 90 J·cm^-2.

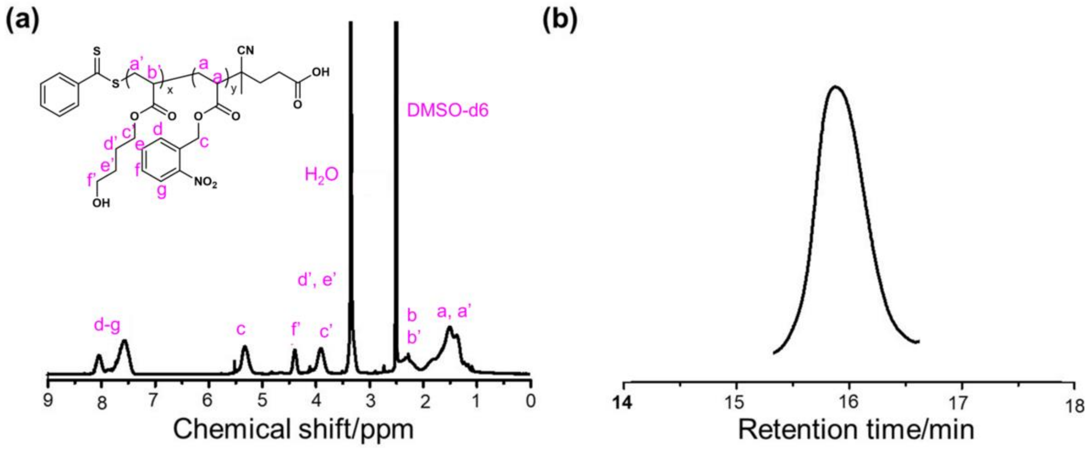

Supplementary Figure 12. ^{1}H NMR spectrum (a) and GPC traces (b) of the poly(HBA-*co*-NBA) obtained.

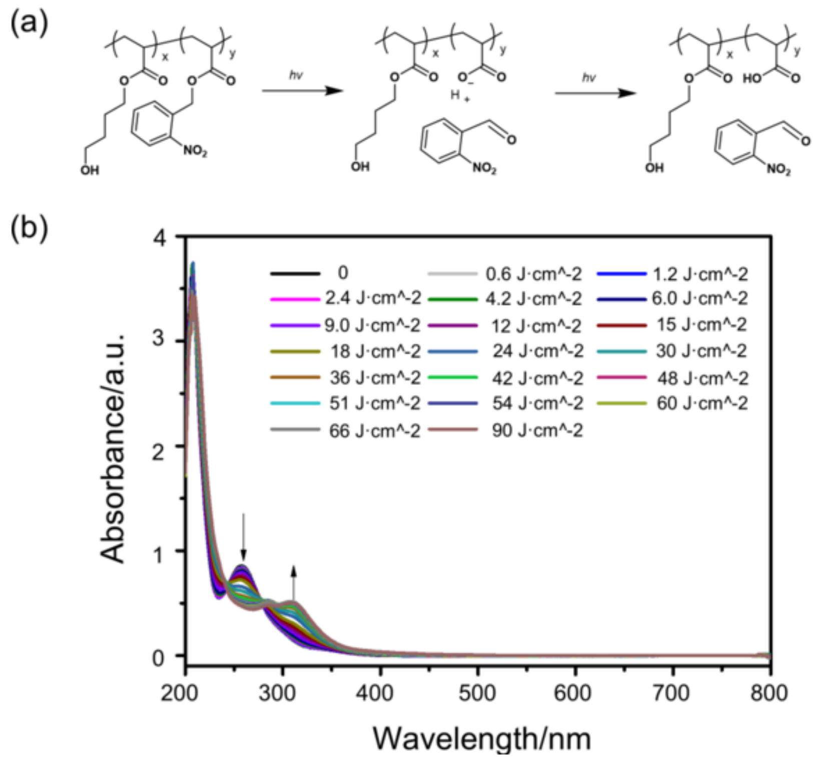

Supplementary Figure 13. Photolysis of poly(HBA-*co*-NBA) copolymers under UV irradiation. (a) Photolytic reaction of the copolymers. (b) Photolysis plot as UV irradiation time increasing. The intensity of the UV light used in this experiment was 10 mW·cm^-2.

Supplementary Note 14: zeta potential of 2-nitrobenzyl alcohol under irradiation

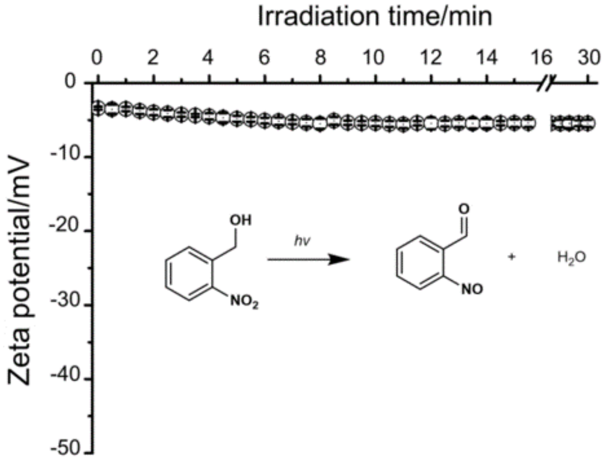

Supplementary Figure 14. Zeta potential of 2-nitrobenzyl alcohol at different irradiation times. A 365 nm LED lamp (10 mW·cm^-2) was used for the irradiation during the measurement.

Supplementary Note 15: Irradiation of control swollen seed-20%

In order to better observe the liquid transportation, the seed-20% was swelled in a solution consisting of HB acetate (97 wt%), I-819 (1 wt%) and BZSA (2 wt%). The swollen seed-20% was subjected to UV irradiation (10 mW·cm^-2). After 30 min irradiation, an obviously bulging was observed in the irradiation region (Supplementary Figure 15a). The surface profile of the irradiated sample was collected immediately (Figure 2d in the main text). After the sample was being stored in dark for 10 hours, the bulged region became subsidence (Supplementary Figure 15b). The surface profile of the irradiated sample was collected again (Figure 2d in the main text).

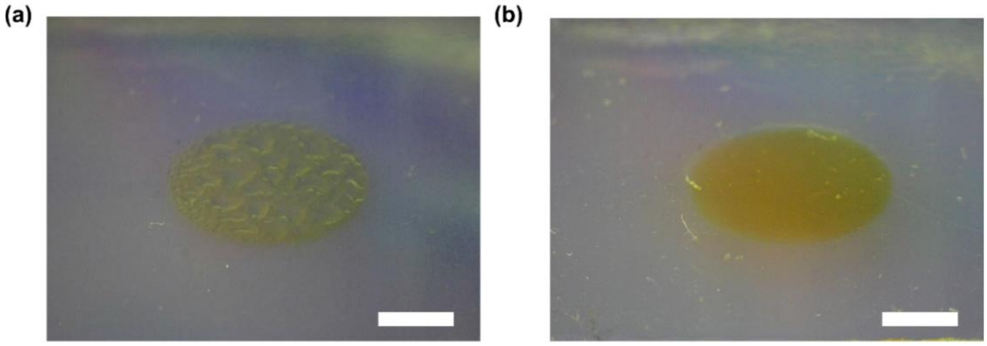

Supplementary Figure 15. Images of the swollen seed-20% after UV irradiation (a) and then storage (b). A solution consisting of HB acetate, I-819 (1 wt%) and BZSA (2 wt%) was used for swelling. Scale bar: 2000 μm.

Supplementary Note 16: calculation of mass transport rate

We evaluated the rate of mass transport in the growth by measuring the diffusion rate of a monomer analogue (4-hydroxylbutyl acetate) under irradiation condition. An analogue was used because HBA monomer will undergo polymerization during irradiation even without any initiator. The polymerization will change the composition of the diffusing liquid. To measure the diffusion rate, a fresh seed-20% sample with a thickness of 500 μm was immersed into 4-hydroxylbutyl acetate and its weight was recorded at different times.

The diffusion rate can be determined using the following supplementary equation (2):[4, 5]

$$F = \frac{M_t - M_0}{M_0} = Kt^n \qquad (2)$$

where F is the rate of diffusion per area; K is a swelling constant, t is the time (s), n is a swelling exponent; M_t and M_0 are the weight of the swollen and dry sample at time t, respectively. From supplementary equation (2), we know that

$$ln F = \ln K + n \ln t \qquad (3)$$

We plotted ln F versus ln t (Supplementary Figure 16) by using the kinetic of swelling yields straight lines up to almost 60% increase in the mass of the swollen sample.[6, 7] The swelling exponents n and the swelling constant K were calculated from the slopes and intercept of the lines. The intercept K

value was used for determination of the diffusion coefficient D:

$$K = 4\sqrt[2]{D/\pi r^2} \quad (4)$$

where D is the diffusion coefficient (cm^2 • s^-1), r is the radius of the cylindrical seed-20% sample (cm).

Combing with the supplementary equation (3), (4) and the plot from Supplementary Figure 16, the diffusion coefficient was 4.7×10^{-5} cm^2·s^-1. As for the control without irradiation, the diffusion coefficient was 4.9×10^{-6} cm^2·s^-1.

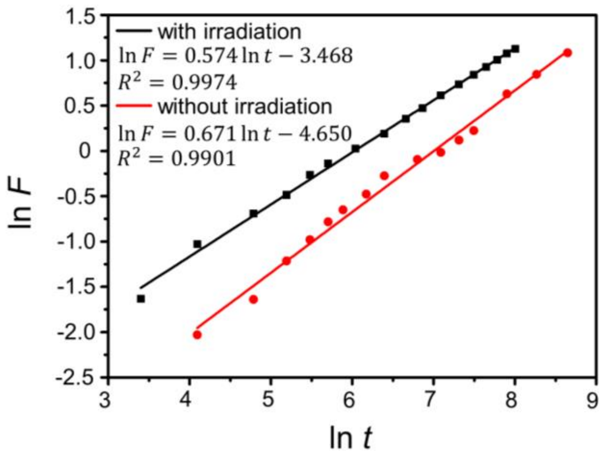

Supplementary Figure 16. Swelling kinetic curve of cylindrical shaped seed-20% samples under different conditions. Samples with a diameter of 1 cm were used.

Supplementary Note 17: change in temperature during growth

An infrared camera was employed to monitor the change in temperature during growth. As shown in Supplementary Figure 17, under our irradiation condition, the temperature can increase from 25 °C to 62 °C after 1 min UV irradiation.

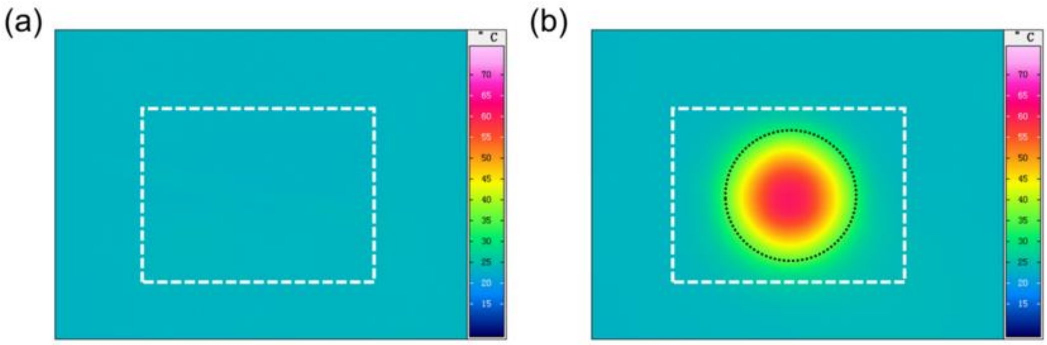

Supplementary Figure 17. Infrared camera image of swollen seed-20% before (a) and after (b) UV irradiation for 1 min. White dotted zone shows the position of swollen sample, while black dotted zone stands for the light irradiation area.

Supplementary Note 18. change in temperature of the sample with non-polymerizable liquids

Under our irradiation condition, there was nearly no increase in the temperature of the irradiated region of swollen seed-20% with non-polymerizable liquids even exposure for 60 min.

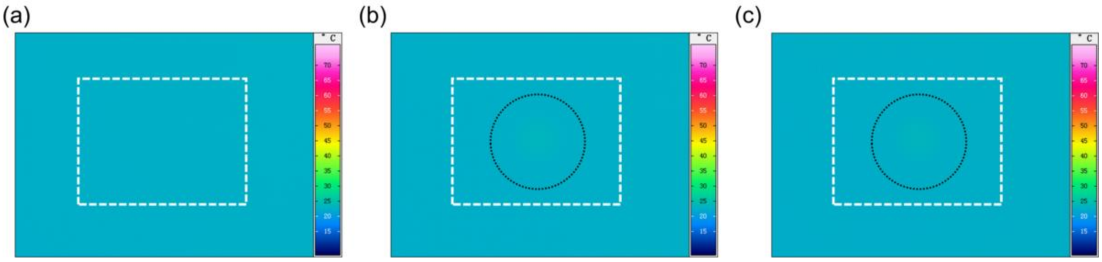

Supplementary Figure 18. Infrared camera image of swollen seed-20% containing non-polymerizable liquids under different conditions: (a) before irradiation, (b) after 1 min irradiation, (c) after 60 min irradiation. A 365 nm LED lamp (10 mW·cm^-2) was used for irradiation. The white dotted zone shows the position of the swollen sample, while the black dotted zone shows the light irradiation area.

Supplementary Note 19: growth of concave structures from seed-20% without BZSA

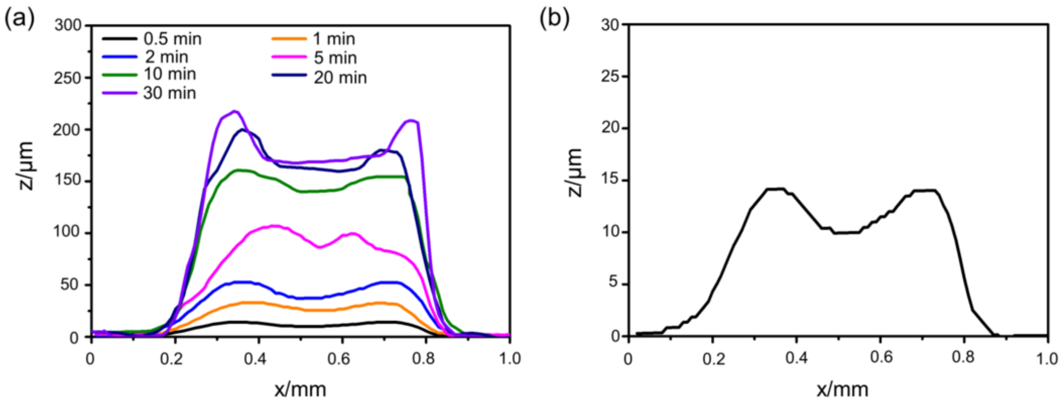

Supplementary Figure 19. Growth height of concave structures of seed-20% without BZSA. (a) Profile of swollen seed-20% without BZSA in the gel matrix at different UV irradiation time. (b) Magnification of black line (0.5 min UV irradiation) in (a).

Supplementary Note 20: formation and homogenization of double network structures

The seed-20% obtained was immersed in the nutrient solutions (containing HBA, HDDA, and I-819) to form swollen seed-20%. During UV irradiation, both the photopolymerization of HBA, HDDA, and I-819 and the photolysis of NBA happened together and a second network formed to generate a double network structure. In such a double network structure, the polymer chains in the original network were stretched, and thus stiffened the materials. Supplementary Figure 20 demonstrates this polymer network structures.

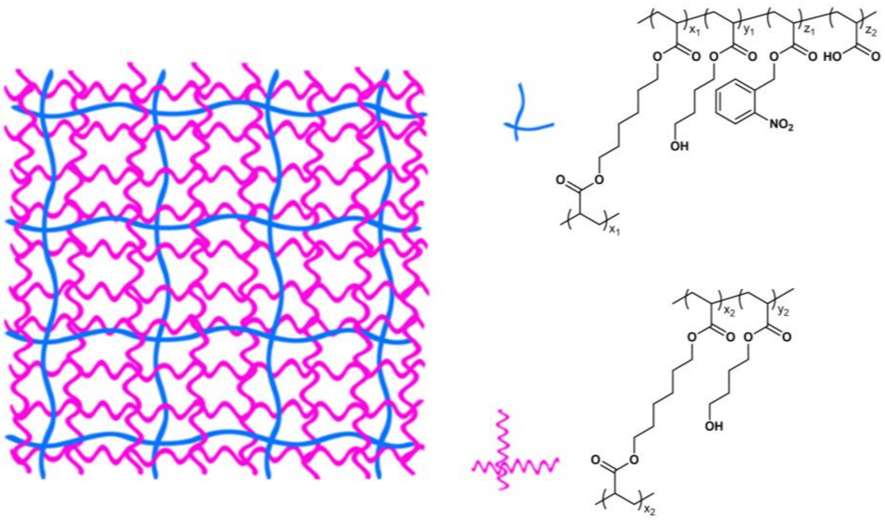

Supplementary Figure 20. Schematic and structures of double network.

In the double network, the polymer chains in the first network was stretched. To release this stress, transesterification was applied to homogenize the structures. BZSA was chosen as the transesterification catalyst due to its relatively low activating temperature (~55 ºC). During UV irradiation, thermal effect generated by the polymerization would induce the transesterification to form homogeneous polymer network of the grown part. Supplementary Figure 21 shows the homogenization mechanism. This homogenization softened the materials (Supplementary Figure 22).

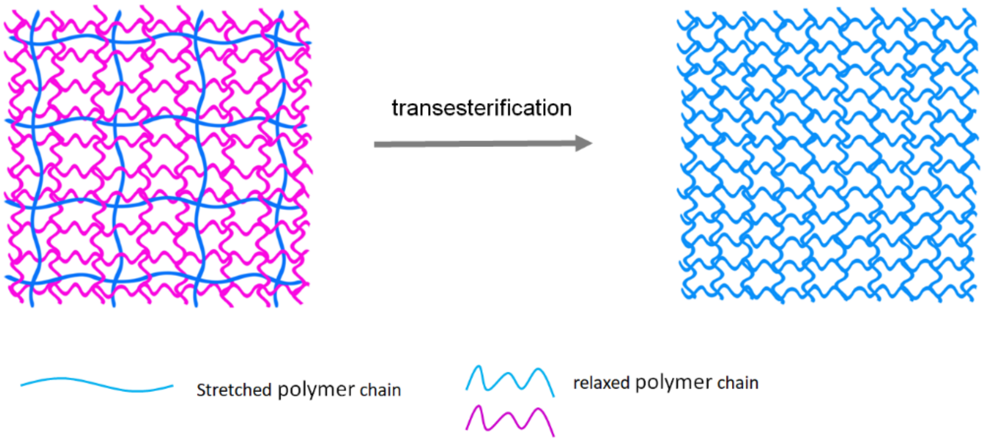

Supplementary Figure 21. Transesterification process in the self-grown part. Transesterification for making homogenous networks.

Supplementary Note 21: modulus of grown structures of HBA-based systems

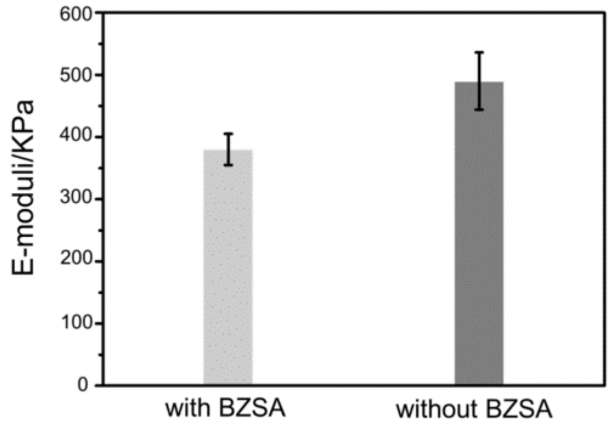

Supplementary Figure 22. E-moduli of the grown structures with and without BZSA as the transesterification catalyst.

Supplementary Note 22: photostability of PDIDA within seed-20% samples

The photostability of the fluorescent molecule (PDIDA) was evaluated by irradiating the dyed seed-20% sample with UV light for 30 min. It was found that the irradiated sample showed almost the same fluorescence spectrum as that of intact sample (Supplementary Figure 23), indicating that the dyed molecule was stable under our irradiation condition.

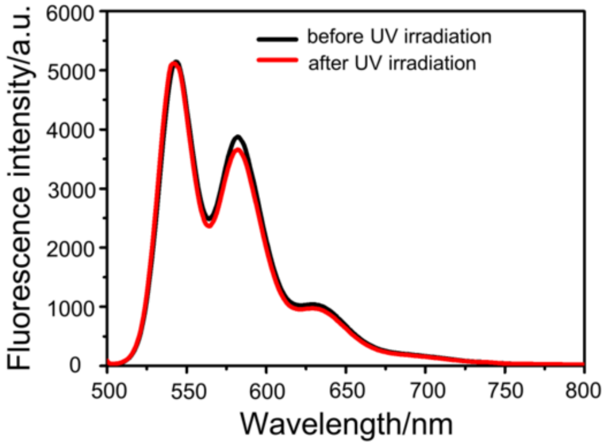

Supplementary Figure 23. Fluorescence spectra of PDI dyed seed-20% thin film before and after UV irradiation.

Supplementary Note 23: fluorescence intensity of dyed seed-20% after growth

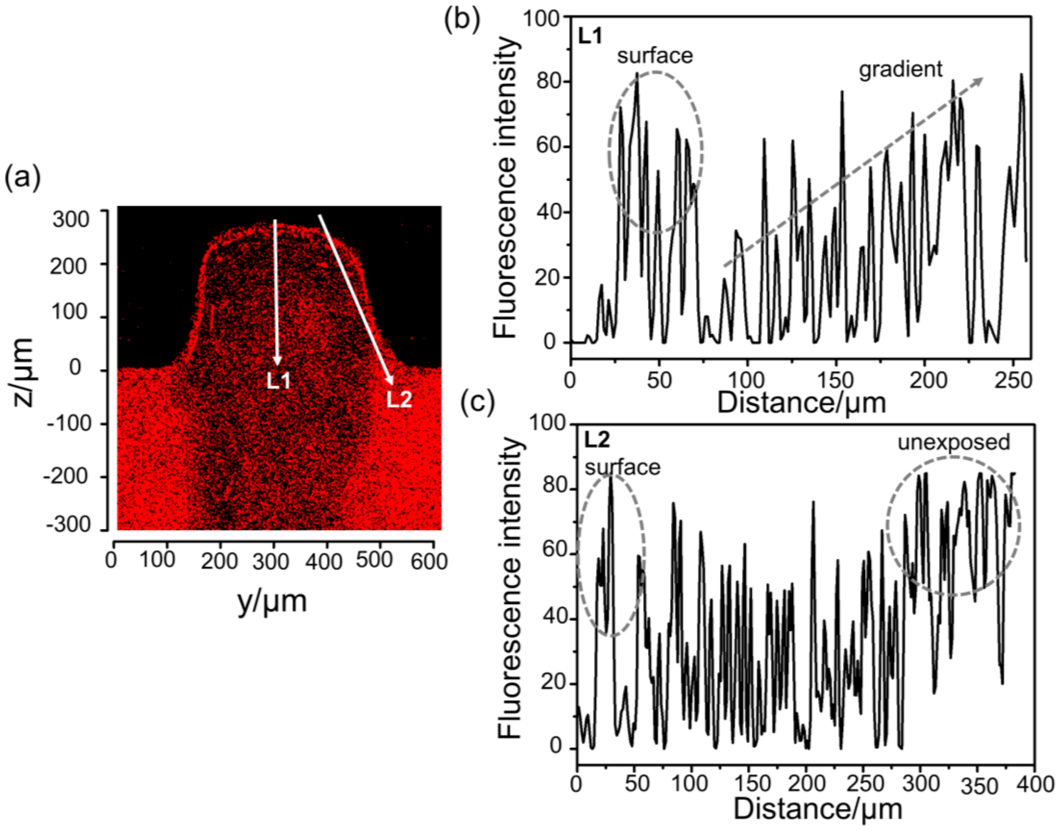

Supplementary Figure 24. Fluorescence intensity of growth structure on dyed seed-20%. (a) Swollen seed-20% dyed by PDIDA in the seed after growth. (b) Fluorescence intensity of L1 in (a). (c) Fluorescence intensity of L2 in (a).

Supplementary Note 24: light-induced growth

Strategy for photo-induced growth

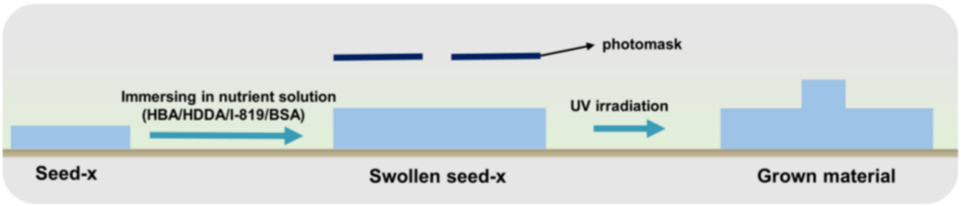

Supplementary Figure 25. Strategy for growth of structure from material seed-x.

Supplementary Figure 25 shows the method used for light-induced growth. A given photomask (a round

shape with diameter of 266 μm and gap of 1000 μm) was placed above the substrate surface with glass slides (thickness: 2 mm) as the spacers. UV 365 nm light with an intensity of 10 mW·cm^-2 was chosen to irradiate the sample through the mask in air. Supplementary Figure 26 shows the SEM image of the grown products.

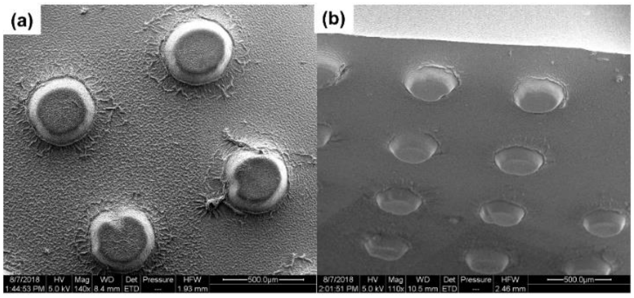

Supplementary Fiugre 26. SEM images of the grown structures. (a) Top view. (b) Side view.

Growth with different crosslinking degree in seed

The protocol was the same as that used for poly(HBA-*co*-NBA) but varying crosslinking fraction. Briefly, precursor solutions containing different HDDA concentrations (0.2 wt%, or 2 wt%, or 5 wt%, or 10 wt%) were used for preparing the seeds. The obtained seeds were immersed in a nutrient solution containing HBA (96 wt%), HDDA (1 wt%), I-819 (1 wt%) and BZSA (2 wt%) to get the swollen seed network for growth. Tensile test was used to measure the E-moduli of the bulky samples with different crosslinker fraction and profilometer was applied to measure the height of the structure.

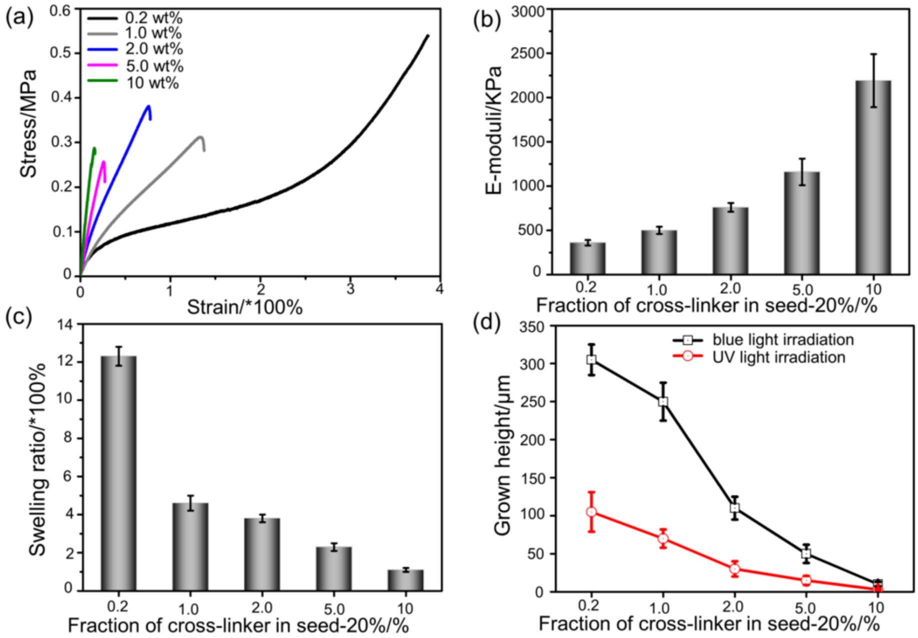

Supplementary Figure 27. Machnical property and grown height of structures on seeds with different crosslinking. (a) Strain-stress curve of seed-20% with different fraction of cross-linkers. (b) E-moduli of samples in (a). (c) Swelling ratio of samples of (a) in nutrient solutions. (d) The height of grown structure at plateau state *vs* different seed-20% samples in (a) under UV light irradiation. Photomask

used in (d) with diameter of 500 μm.

Growth with different photomasks

Photomasks with various scales (round shapes with diameter from 266 μm to 5000 μm) were utilized to control the irradiation area. After exposed to UV light until the samples did not grow anymore, the grown heights of the structures were measured from profiles.

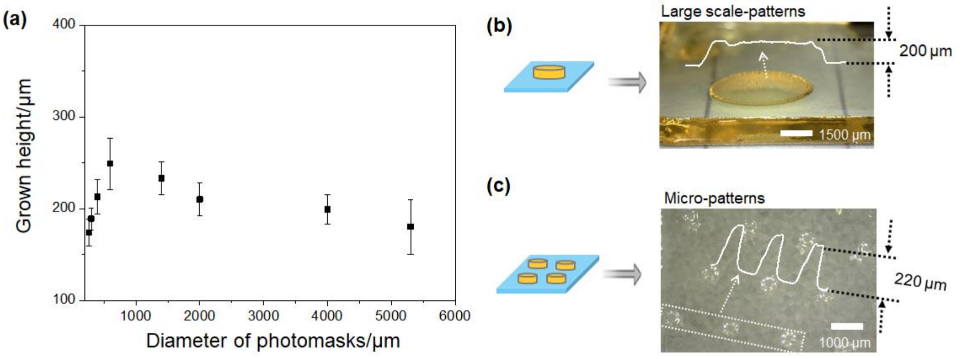

Supplementary Figure 28. Grown height of structures on seed-20% with different photomasks. (a) The height of grown structure at plateau state versus diameter of photomask under UV light for 30 min. (b) Grown large scale pattern with a diameter of 4000 μm. The height of this pattern was 200 μm. (c) Grown micro-pattern with a diameter of 400 μm. The height of this pattern was 220 μm. The white lines in (b) and (c) were the profiles of the grown structures.

Growth under different light intensities

The swollen seed-20% prepared above was irradiated under UV 365 nm light with different light intensities (from 0.1 mW·cm^-2 to 13 mW·cm^-2) for 30 min.

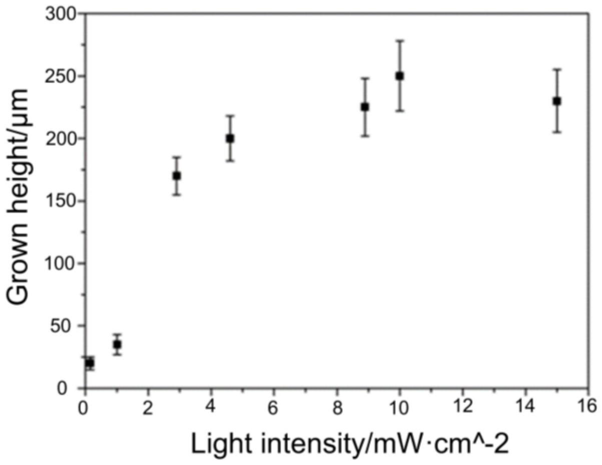

Supplementary Figure 29. The height of grown structure after 30 min irradiation versus light intensity. The diameter of the activated round hole from photomask was 500 μm.

Poly(PEGA-*co*-NBA) systems

The protocol was the same as that used for poly(HBA-*co*-NBA) but replacing HBA with PEGA. Briefly, PEGA (80% molar ratio), NBA (20% molarl ratio), HDDA (1 wt%) and I-819 (1 wt%) are mixed together and used for preparing the seeds. This obtained seed network was immersed in a nutrient solution containing PEGA, HDDA, I-819 and BZSA to get the swollen PEGA based seed network for growth. Profilometer was applied to measure the height of the structure and indentation was used to measure the E-moduli of the materials.

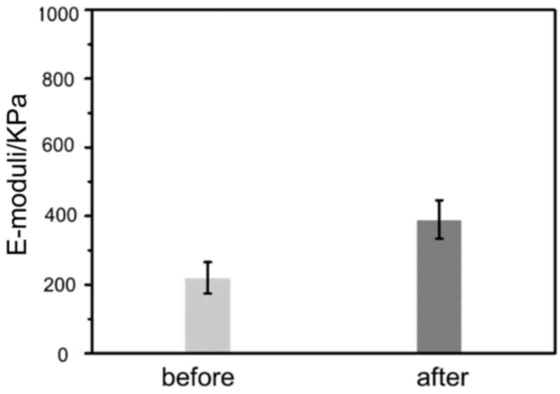

Supplementary Figure 30. E-moduli of PEGA based seed before and after growth.

Poly(BA-*co*-NBA) systems

The protocol was the same as that used for poly(HBA-*co*-NBA) but replacing HBA with BA. Profilometer was applied to measure the height of the structure and indentation was used to measure the E-moduli of the materials.

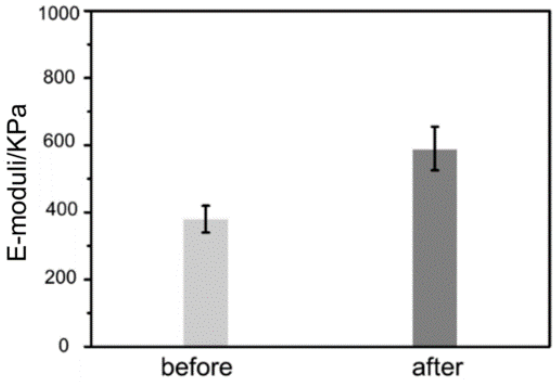

Supplementary Figure 31. E-moduli of BA based seed before and after growth.

Hybrid systems

A PEGA-based seed 20% was used for growth. It was immersed in a nutrient solution containing HBA,

HDDA, I-819, and BZSA to get the swollen seed. Then UV 365 nm light with an intensity of 10 mW·cm^-2 was used to trigger the growth of the hybrid structures. Profilometer was applied to measure the height of the structure and indentation was used to measure the E-moduli of the materials.

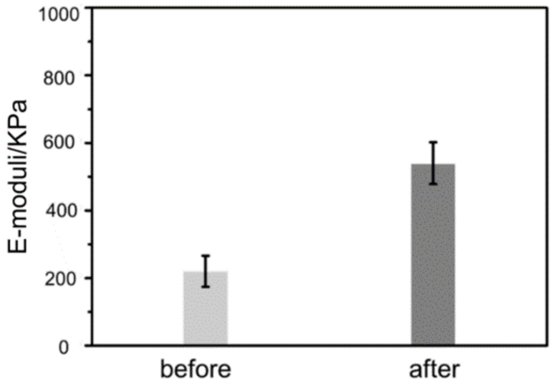

Supplementary Figure 32. E-moduli of hybrid PPEGA-PHBA based materials before and after growth.

Sequential growth of structures on material surfaces

A HBA-based seed-20% was used. A big structure (a round shape with a diameter of 5000 μm) was grown out from the swollen sample under UV irradiation for 20 min. After the growth, the sample was immersed in ethanol solution to remove the unreacted monomers, and dried in air. This sample obtained was re-swelled in the nutrient solution to obtain the second swollen seed-20%. Finally, the second swollen seed networks were irradiated with UV light through a photomask with a diameter of 1250 μm for 30 min.

Growth with different nutrient solutions

The HBA-based seed-20% were used. They were swelled in nutrient solutions containing different content of crosslinkers, such as 0.2%, 2%, 5% and 10% to attain the swollen seed-20%, followed by being exposed to the UV light with suitable photomasks for 30 min. Indentation was used to measure the E-moduli of the grown structures of the material surfaces.

Supplementary Note 25: microstructures made by UV laser

HBA-based seed-20% were used. They were swelled in nutrient solutions consisting of HBA, 1 wt% HDDA, 1 wt% I-819, 2 wt% BZSA and 0.01 wt% PDIDA, followed by subjecting to primo microscopy equipped with 365 nm UV laser for writing. Models with different shapes were adopted, such as alphabet (INM), square domains and Chinese Tai Chi, and the UV laser would only emit in the white region shown in Supplementary Figure 33a. To make patterns on the substrate surface, the intensity of the UV laser was set as 1V with an exposure time of every 30s. After illumination, the samples were rinsed with

ethanol/$CHCl_3$ before analyzing with confocal microscopy. Supplementary Figure 33b shows the optical image of different microstructures embossed from the samples. Well-defined microstructures were obtained.

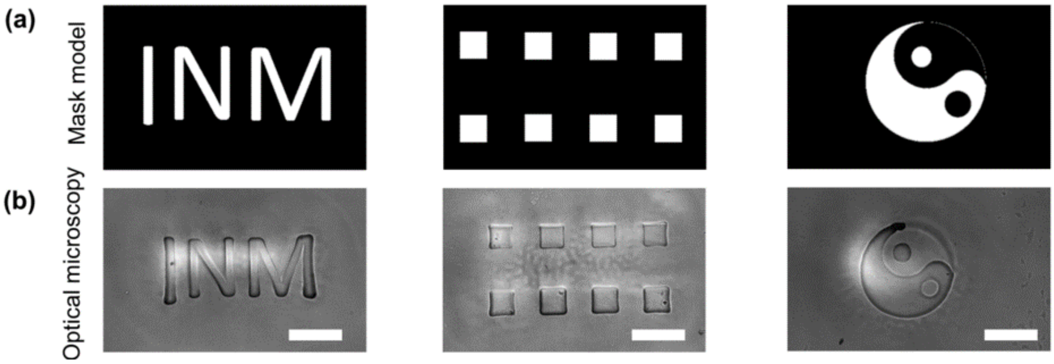

Supplementary Figure 33. Laser writable microstructures on the substrate surface. (a) Mask model used in the primo experiments. (b) Optical microscopy of the writable patterns. Scale bar in the pattern of the alphabet (INM), square domains and Chinese Tai Chi is 100 μm.

Supplementary Note 26: restoration of large damage

An unregular damaged region was created on HBA-based seed-20% by adding a piece of unregular glass slide in the seed solution before photopolymerization. The obtained seed-20% with the damaged region was immersed in a nutrient solution containing HBA, HDDA, I-819, and BZSA to afford swollen seed-20%. A photomask with a diameter of 3.6 mm was put above the material surface, and 365 nm UV light was used to trigger the growth to self-restore the materials. The pictures were recorded by a camera. The 3D profiles and surface profile were collected by a profilometer.

Supplementary References

1. Lai, J. *et al.* Light-responsive nanogated ensemble based on polymer grafted mesoporous silica hybrid nanoparticles. *Chem. Commun.* **46**, 7370-7372 (2010).

2. Desrat, S., Remeur, C. & Roussi, F. Development of an efficient route toward meiogynin A-inspired dual inhibitors of Bcl-xL and Mcl-1 anti-apoptotic proteins. *Org. Biomol. Chem.* **13**, 5520-5531 (2015).

3. Zhu, L. *et al.* Reversibly photoswitchable dual-color fluorescent nanoparticles as new tools for live-cell imaging. *J. Am. Chem. Soc.* **129**, 3524-3526 (2007).

4. Karadağ, S.; Üzüm, Ö & Saraydin, D. Swelling equilibria and dye adsorption studies of chemically crosslinked superabsorbent acrylamide/maleic acid hydrogels. *Eur. Polym. J.* **38**, 2133-2141 (2002).

5. Yiamsawas, D. *et al.* Synthesis and swelling properties of poly [acrylamide-*co*-(crotonic acid)] superabsorbents. *React. Funct. Polym.* **67**, 865-882 (2007).

6. Pappas, N. & Franson, N. The swelling interface number as a criterion for prediction of diffusional

solute release mechanisms in swellable polymers. *J. Polym. Sci.: Polym. Phys. Ed.* **2**1, 983-997 (1983).

7. Jabbari, E. & Nozari, S. Swelling behavior of acrylic acid hydrogels prepared by γ-radiation crosslinking of polyacrylic acid in aqueous solution. *Eur. Polym. J.* **36**, 2685-2692 (2000).
